# Patient‐derived xenografts: Practical considerations for preclinical drug testing

**DOI:** 10.1002/hem3.70133

**Published:** 2025-04-16

**Authors:** Charles E. de Bock

**Affiliations:** ^1^ Lowy Cancer Research Centre, Children's Cancer Institute Randwick New South Wales Australia; ^2^ Faculty of Medicine UNSW, School of Clinical Medicine Sydney New South Wales Australia

Patient‐derived xenografts (PDXs) are increasingly being used to test new therapies or repurpose existing therapies as researchers and clinicians optimize precision oncology treatments.^1^ This has been further accelerated with the increasing availability of new immunodeficient mice that have improved our ability to generate a wider variety of PDXs, including for challenging leukemia subtypes such as favorable risk acute myeloid leukemia (AML). Inspired by the conversation with Prof Richard Lock who features in a *HemaSphere* podcast reflecting on over 20 years of experience in preclinical testing,^2^ this article reflects some of the practical considerations for establishing a PDX bank and their use in evaluating new therapies.

## ESTABLISHING AND MAINTAINING PDXS OVER THE LONG TERM

Immunodeficient mice provide the opportunity to engraft human leukemia cells and generate a PDX model. These are the models of systemic disease that infiltrate the bone marrow, spleen, and liver and disseminate throughout the peripheral blood. They are attractive models because they retain the cellular and molecular characteristics of the original disease with leukemia burden monitored through peripheral blood sampling or via bioluminescence.

**Figure 1:**
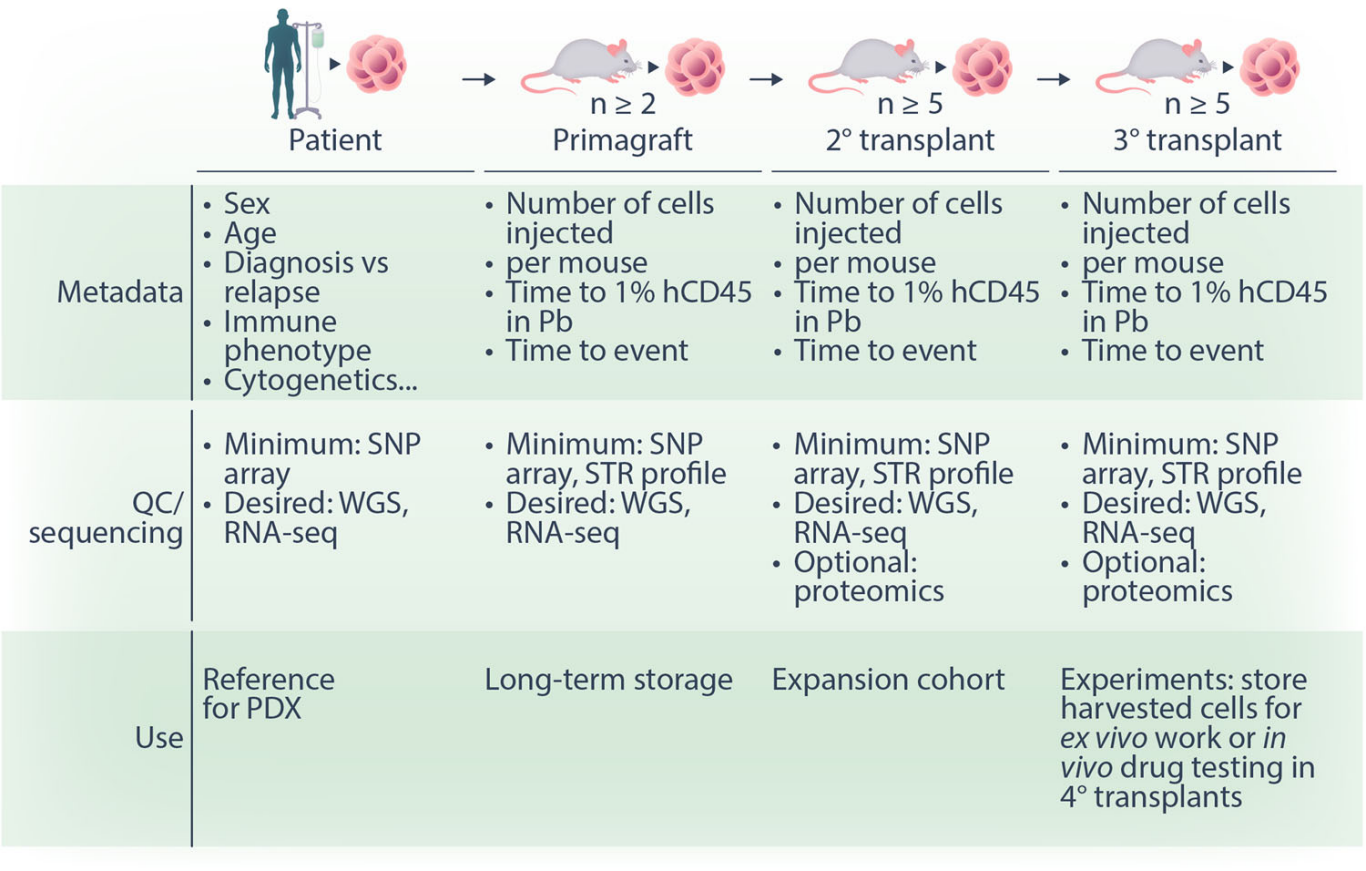
**Schematic outlining the process of establishing a single PDX**. This includes the original primagraft and secondary transplants that should be protected for long‐term storage and quality assurance. Cells from the tertiary transplants are recommended for downstream applications. Molecular characterization and sequencing should be routinely carried out, and while the patient sample can serve as a reference for the PDX, downstream applications should be based on the clonal composition and genetic heterogeneity of the PDX sample.

To establish a PDX, patient cells are injected into the tail vein or intrafemorally of immunodeficient mice (Figure 1). This first round of engraftment or primagrafts usually has the slowest kinetics of engraftment time depending on the quality and source of the patient sample. Once leukemia develops in these primagrafts, the cells can be harvested from highly engrafted mice (e.g., human CD45+ve cells > 80% in the peripheral blood) and serially reinjected into secondary and tertiary recipients after which the kinetics of engraftment stabilises and is usually consistent across multiple transplants.

Importantly, when establishing new PDX samples, it is recommended that cells harvested from primagraft, and secondary transplant cells are protected and stored over the long term with only cells from tertiary transplants used in downstream experiments. This will ensure the longevity of the PDX bank and provide an important reference for quality assurance regarding clonal and genetic heterogeneity.

Alongside the technical establishment of the PDX, ensuring excellent record keeping (e.g., the time taken to reach 1% human CD45 cells in the peripheral blood) and adhering to the published minimum information standards for PDX models is important for the field in terms of reproducibility and sharing of PDX resources.^3^ This includes metadata on the original patient sample and sequencing methodology for the molecular characterization of the PDX (Figure 1). This characterization of the PDX is essential for downstream preclinical drug testing when individual samples are chosen based on the expression or a biomarker or the presence of a genetic mutation.

It is equally important that PDX samples used in downstream experiments are routinely checked (i.e., using single‐nucleotide polymorphism arrays or whole genome sequencing) and referenced back to the primagraft and secondary transplants similar to the routine short tandem repeat profiling of cell lines. This characterization will quickly identify any mislabeled samples and ensure the long‐term integrity of the PDX bank.

## WHICH IMMUNE‐COMPROMIZED MOUSE MODEL IS BEST FOR YOUR STUDY?

The most common immunodeficient mouse used in contemporary PDX generation is the NOD.Cg‐Prkdcscid Il2rgtm1Wjl/SzJ (NSG) mouse that is also referred to as NOD‐*scid* IL2Rgamma^null^, NOD‐*scid* IL2Rg^null^, or NOD *scid* gamma. These mice have no functional T cells and B cells and no natural killer cells, which allows the efficient engraftment of human cells.^4^ This strain has been useful for engraftment of lymphoid malignancies but more challenging for myeloid malignancies. Improved engraftment rates of AML samples have been achieved using NSG‐SGM3 (NSG humanized with SCF, GM‐CSF, and IL‐3) including cases where cells were from intermediate‐risk AML patients. Interestingly, this study also found that sex was a variable for engraftment with male patients agnostic to sex of recipient mice but female patients generated significantly higher engraftment into female.^5^

A new commercial immunodeficient mouse strain that can assist with AML PDX engraftment is the MISTRG (M‐CSFh/h IL‐3/GM‐CSFh/h hSIRPh/h TPOh/h Rag2^−/−^ Il2rg^−/−^) mouse that has human IL‐3, GM‐CSF, TPO, and M‐CSF genes knocked‐in to replace their murine counterparts, thereby expressing physiologically relevant levels of these cytokines. Importantly, these mice support the engraftment and maintenance of leukemia‐initiating cell (LIC) cells, which have implications in testing new therapies designed to target this cell population.^6^ However, these mice can be difficult to obtain; therefore, another alternative is the NBSGW strain generated by crossing NSG mice with C57BL/6J‐KitW‐41J/J (C57BL/6.KitW41) mice is also permissive to AML engraftment even in the absence of preconditioning with ionizing radiation.^7^

## USING OBJECTIVE RESPONSE MEASURES FOR TREATMENT RESPONSE IN PRECLINICAL TESTING

One of the major challenges in preclinical testing is when to start treatment and when to assess event‐free survival (EFS) when death is not considered an ethical endpoint. Furthermore, researchers are rarely blinded to the treatment mice receive, making it essential that an unbiased assessment of endpoint is applied consistently across different experiments and researchers. As explained by Professor Richard Lock, within the preclinical testing consortium, drug treatments begin when mice reach 1% human CD45 in the peripheral blood. The mice are then monitored once per week via peripheral blood draw for the duration of the study period, and objective response measures (ORM) are used to assess drug efficacy (Table 1). These measures were established in part due to the immense success rate of standard‐of‐care chemotherapy with new compounds that only result in progressive or stable disease irrespective of a statistical difference in EFS unlikely to be used clinically.

**Table 1:** Objective response measure for ALL PDX models.

Response	Definition
Progressive disease (PD)^a^	Human CD45% never drops below 1% in the peripheral blood and mouse reaches event (human CD45% >25%) during study period.
Stable disease (SD)	Human CD45% never drops below 1% and mouse never reaches event by the end of the study period.
Partial response (PR)	Human CD45% drops below 1% at least once during study period, but not CR.
Complete response (CR)	Human CD45% drops below 1% for at least two consecutive weekly readings during the study period regardless of whether the event is reached at a later time point.
Maintained complete response (MCR)	Human CD45% drops below 1% for at least three consecutive weekly readings at any time after treatment has been completed.

a PD can be further classified into two separate categories based on leukemia growth delay; please see Randall et al.^8^ for comprehensive definitions.

However, when researchers are establishing proof of principle efficacy using tool compounds, these ORM might not be appropriate and statistical differences in EFS sufficient to assess efficacy. Nevertheless, researchers should still record and report on the kinetics of leukemia burden during and after treatment and have a predetermined event cut‐off to calculate EFS (e.g., 25% human CD45 in the peripheral blood) that will help produce robust data for assessing drug efficacy.

## CONVENTIONAL VERSUS SINGLE MOUSE TRIAL (SMT) FORMATS FOR IN VIVO PRECLINICAL TESTING

**Figure 2:**
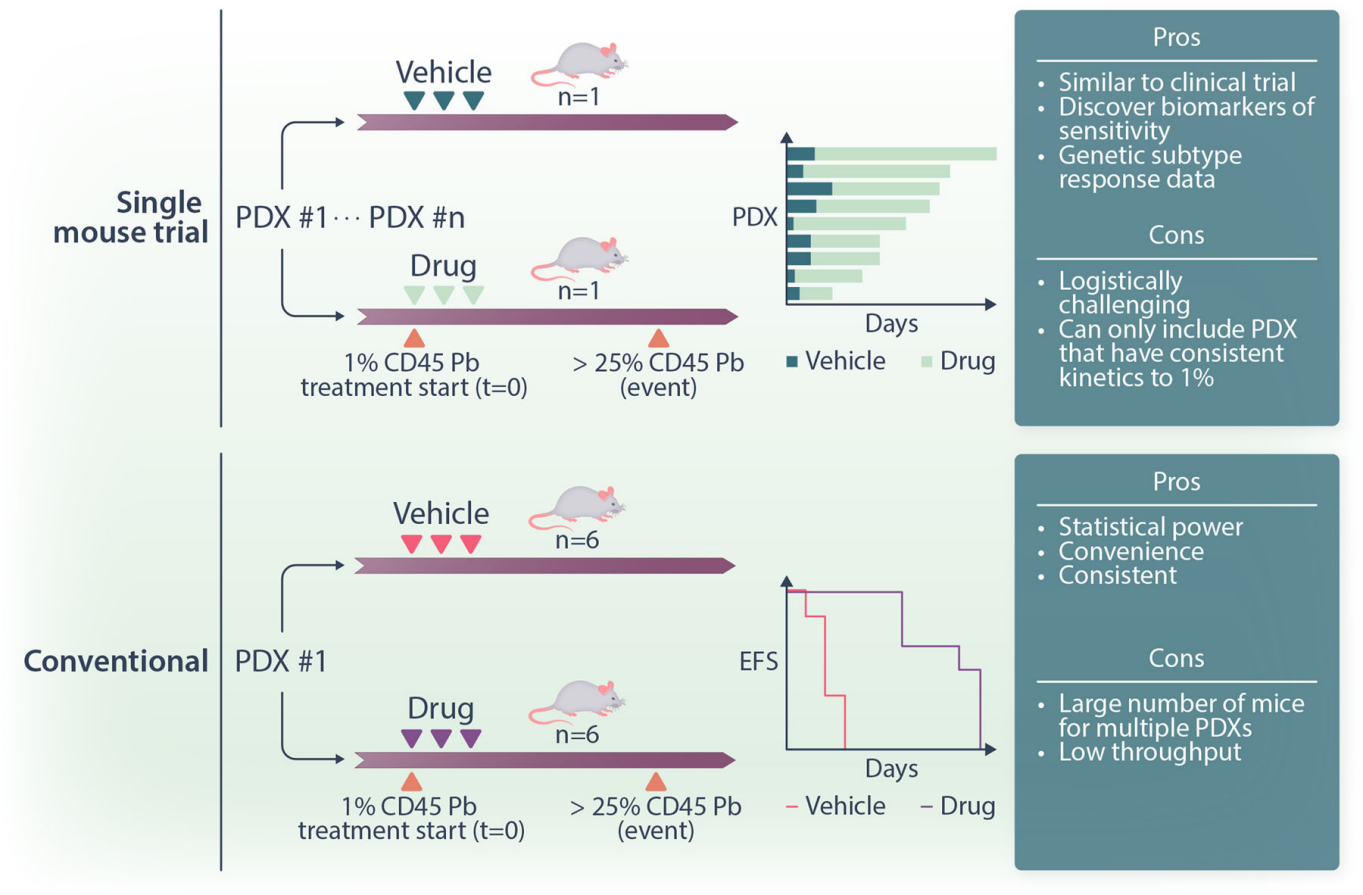
**Schematic comparing conventional and single mouse trial formats for preclinical drug testing**.

The assessment of targeted therapies is conventionally tested in a PDX model with each arm of the treatment regimen having approximately six mice. Treatment can then be compared to vehicle control and differences in EFS determined and charted using Kaplan–Meier survival curves (Figure 2). An alternative to this conventional method is the SMT format, which is recommended for assessing the efficacy of a drug across a large set of PDXs.^8^ The SMT allows response to be measured across diverse genetic subtypes of leukemia and can identify associated biomarkers. Another advantage of the SMT is the reduction of mouse numbers with a recent study assessing the CD123 antibody–drug conjugate pivekimab sunirine requiring a total of 78 mice to assess efficacy across 39 PDX models.^9^ This is in contrast to a conventional study assessing a second‐generation proteasome inhibitor ixazomib in eight different T‐ALL PDX samples that used 128 mice.^10^ However, SMTs can be logistically challenging to run and requires PDX samples with consistent leukemia growth kinetics (Figure 2).

## CONCLUDING REMARKS

PDX models continue to play a central role in understanding both leukemia biology and assessing novel agents. However, to quote the British statistician George Box, “All models are wrong, but some are useful” holds true for PDX models and their limitations should be recognized. Immunodeficient mice are, for example, very sensitive to anthracyclines due to the *scid* mutation or conversely can tolerate doses that are clinically unachievable. Similarly, establishing PDX models is only possible due to the lack of an adaptive immune system in host mice; therefore, interactions and impacts of a treatment on normal B‐ and T cells can be difficult to determine. Therefore, appreciating both the advantages and the limitations of the PDX model alongside standardized reporting in assessing efficacy will improve the development and translation of new therapies into the clinic.

## AUTHOR CONTRIBUTIONS

Charles E. de Bock conceptualized and wrote the article.

## CONFLICT OF INTEREST STATEMENT

The author declares no conflicts of interest.

## FUNDING

No funding was received for this publication.

## Data Availability

Data sharing is not applicable to this article as no datasets were generated or analyzed during the current study.

## References

[1] Blanchard Z , Brown EA , Ghazaryan A , Welm AL . PDX models for functional precision oncology and discovery science. Nat Rev Cancer. 2025;25(3):153‐166. 39681638 10.1038/s41568-024-00779-3 PMC12124142

[2] Smith MA , Houghton PJ , Lock RB , et al. Lessons learned from 20 years of preclinical testing in pediatric cancers. Pharmacol Ther. 2024;264:108742. 39510293 10.1016/j.pharmthera.2024.108742 PMC12085791

[3] Meehan TF , Conte N , Goldstein T , et al. PDX‐MI: minimal information for patient‐derived tumor xenograft models. Cancer Res. 2017;77(21):e62‐e66. 29092942 10.1158/0008-5472.CAN-17-0582 PMC5738926

[4] Zhou Q , Facciponte J , Jin M , Shen Q , Lin Q . Humanized NOD‐SCID IL2rg–/– mice as a preclinical model for cancer research and its potential use for individualized cancer therapies. Cancer Lett. 2014;344(1):13‐19. 24513265 10.1016/j.canlet.2013.10.015

[5] Mian SA , Ariza‐McNaughton L , Anjos‐Afonso F , et al. Influence of donor‐recipient sex on engraftment of normal and leukemia stem cells in xenotransplantation. HemaSphere. 2024;8(5):e80. 38774656 10.1002/hem3.80 PMC11107397

[6] Connerty P , Xie J , El‐Najjar F , et al. Immune‐deficient MISTRG mice support expansion of leukaemia‐initiating cells in xenograft models of paediatric acute myeloid leukaemia. Br J Haematol. 2025;206(4):1025‐1255. 10.1111/bjh.20029 39984432

[7] Dembitz V , Durko J , Campos J , et al. Immunodeficient NBSGW mouse strain allows chemotherapy modeling in AML patient‐derived xenografts. HemaSphere. 2024;8(1):e28. 38434525 10.1002/hem3.28 PMC10878184

[8] Murphy B , Yin H , Maris JM , et al. Evaluation of alternative in vivo drug screening methodology: a single mouse analysis. Cancer Res. 2016;76(19):5798‐5809. 27496711 10.1158/0008-5472.CAN-16-0122 PMC5050128

[9] Watts B , Smith CM , Evans K , et al. The CD123 antibody‐drug conjugate pivekimab sunirine exerts profound activity in preclinical models of pediatric acute lymphoblastic leukemia. HemaSphere. 2025;9(1):e70063. 39830370 10.1002/hem3.70063 PMC11739898

[10] Randall J , Evans K , Watts B , et al. In vivo activity of the second‐generation proteasome inhibitor ixazomib against pediatric T‐cell acute lymphoblastic leukemia xenografts. Exp Hematol. 2024;132:104176. 38320689 10.1016/j.exphem.2024.104176 PMC10978271

